# Sober Curiosity: A Qualitative Study Exploring Women’s Preparedness to Reduce Alcohol by Social Class

**DOI:** 10.3390/ijerph192214788

**Published:** 2022-11-10

**Authors:** Belinda Lunnay, Emily Nicholls, Amy Pennay, Sarah MacLean, Carlene Wilson, Samantha B. Meyer, Kristen Foley, Megan Warin, Ian Olver, Paul R. Ward

**Affiliations:** 1 Research Centre for Public Health, Equity and Human Flourishing, Torrens University, Adelaide 5000, Australia; 2 Department of Sociology, University of York, York YO10 5DD, UK; 3 Centre for Alcohol Policy Research, La Trobe University, Melbourne 3000, Australia; 4 College of Medicine and Public Health, Flinders University, Bedford Park 5042, Australia; 5 School of Public Health Sciences, University of Waterloo, Waterloo, ON N2L 3G1, Canada; 6 Fay Gale Centre for Research on Gender, School of Social Sciences, University of Adelaide, Adelaide 5000, Australia; 7 School of Psychology, University of Adelaide, Adelaide 5000, Australia

**Keywords:** sober curious movement, sober curiosity, alcohol reduction, drinking culture, women, alcohol, midlife, middle age, social class

## Abstract

Background: Urgent action is required to identify socially acceptable alcohol reduction options for heavy-drinking midlife Australian women. This study represents innovation in public health research to explore how current trends in popular wellness culture toward ‘sober curiosity’ (i.e., an interest in what reducing alcohol consumption would or could be like) and normalising non-drinking could increase women’s preparedness to reduce alcohol consumption. Methods: Qualitative interviews were undertaken with 27 midlife Australian women (aged 45–64) living in Adelaide, Melbourne and Sydney in different social class groups (working, middle and affluent-class) to explore their perceptions of sober curiosity. Results: Women were unequally distributed across social-classes and accordingly the social-class analysis considered proportionally the volume of data at particular codes. Regardless, social-class patterns in women’s preparedness to reduce alcohol consumption were generated through data analysis. Affluent women’s preparedness to reduce alcohol consumption stemmed from a desire for self-regulation and to retain control; middle-class women’s preparedness to reduce alcohol was part of performing civility and respectability and working-class women’s preparedness to reduce alcohol was highly challenging. Options are provided for alcohol reduction targeting the social contexts of consumption (the things that lead midlife women to feel prepared to reduce drinking) according to levels of disadvantage. Conclusion: Our findings reinstate the importance of recognising social class in public health disease prevention; validating that socially determined factors which shape daily living also shape health outcomes and this results in inequities for women in the lowest class positions to reduce alcohol and related risks.

## 1. Introduction

Alcohol consumption remains a major public health problem, contributing to myriad preventable conditions and increasing the risk of violence. Management of adverse outcomes of alcohol is estimated to cost Australia $66.8 billion a year [1]. As such, Australia’s National Alcohol Strategy 2019–2028 aims for a 10% reduction in harmful population-level alcohol consumption [2]. However, reduction messages need to account for the fact that alcohol consumption at risky levels is socially acceptable particularly for heavy drinking sub-populations, including women in midlife aged 45–64 years (herein defined as ‘midlife women’) [3,4] despite current Australian Guidelines which recommend drinking no more than two standard drinks on any day to reduce the lifetime risk of harm from alcohol-related disease or injury, and no more than four standard drinks per occasion to reduce short-term acute harms. In 2019, the heaviest drinking 10% of the Australian population accounted for 54.1% of all alcohol consumed [5]. Central to this paper, midlife women in particular are at a higher risk of lifetime harm than other sub-populations of Australians, and while men consume more alcohol than women (at a population level) alcohol causes more physiological harms to women because of differences in metabolism compared to men [6]. Patterns of alcohol consumption at this age (45–64 years) have been trending up (while drinking among other sub-population groups such as young Australians is trending down). Midlife women are thus, a target group identified within the National Alcohol Strategy as warranting urgent intervention. In the 2020s, women are drinking more alcohol than previous generations of women in this stage of life, and more than any other age group currently, and the reasons for this lie in research which indicates consumption provides some women with a form of stress-relief [7] and self-care [8,9,10] that is socially acceptable [11,12] and can function as a tool to promote wellness, sometimes within a limited range of resources; and women’s options diminish as they experience more social disadvantage [13]. Australian women’s reasons for alcohol consumption are also differentiated on the basis of social class [13,14,15]. Of particular relevance to this study is that affluent women have more agentic ‘relationships’ with alcohol, whereas there is a tendency for less control over alcohol-related decisions for women living with less privilege. This prompts a question about how differences in social class translate to women’s capabilities for alcohol reduction—strategies might be less readily accessible to women from lower social classes in order to reduce consumption, than those for women with more resources.

Urgent action is required to identify socially acceptable alcohol reduction options for heavy drinking midlife Australian women. The emerging evidence of widespread curiosity among middle-aged woman about sobriety and alcohol reduction highlights a messaging tactic that may provide a new public health campaign strategy for harm reduction. Herein we engage with the notion of sober curiosity; that is, for some, a socially acceptable movement challenging the idea that specific social contexts require alcohol. While the reasons for midlife women’s alcohol consumption are well explored; there is a gap in knowledge about options, like sober curiosity, that might enable sustained reductions in consumption among this heavy drinking sub-population. Further, given the complex structural factors that may make it difficult (and perhaps even impossible) for some midlife women to contemplate reducing alcohol consumption, it is important to explore how these movements and ‘options’ may vary with social class. Nonetheless, the feasibility of reducing midlife women’s alcohol consumption while the concept of ‘sober curiosity’ is in the public spotlight offers new opportunities for public health in contrast to typical approaches that use instruction on limiting alcohol intake as a guiding principle. The study reported here represents a paradigm shift where the normative social environment supports moderate or no intake. In this paper we present our findings and explore factors that impact women’s preparedness to reduce drinking including, what women anticipate gaining or losing from consuming less alcohol or not drinking at all and important considerations in designing messages that reinforce sober curiosity.

## 2. The ‘Sober Curious’ Movement and Reducing Alcohol Consumption

The sober curious movement has developed progressively over the past decade and a half but has become highly prominent recently among social media influencers (mostly women and young people) who pitch not drinking or drinking in moderation (i.e., framed as drinking ‘mindfully’) as pleasurable and beneficial. Part of the ‘curiosity’ of the movement entails exploring the idea that social contexts generally associated with alcohol use can be enjoyable without it, thus challenging social norms that position drinking as the ‘default’ position in many social settings. Sober curiosity differs from complete abstinence (‘quitting alcohol’); the latter is supported by popular and well-known organised periods of non-drinking such as ‘Dry July’ or ‘FebFast’ that are usually short-term (lasting one month) and validated through a philanthropic pursuit such as raising money for a cause. Sober curiosity is geared toward moderating consumption and decreasing risky drinking practices in a manner that is sustainable over the long-term; encouraging an ongoing ‘questioning’ of drinking alcohol and a decision to reflect on reasons for drinking relative to alcohol-related health risks. Whilst a period of sober curiosity may ultimately lead to a decision to stop drinking completely, the emphasis is primarily on reflection—and subsequent change—rather than necessarily on complete abstinence. The movement is a shift away from binary conceptualisations of ‘normal’ versus ‘problem’ drinking that might associate particular drinking practices with addiction and advocate full abstinence only (as evidenced through 12-step programs such as Alcoholics Anonymous) [16]. Various other approaches to reduce alcohol consumption are possible including restrictions on availability for example through price control measures. However, with the exception of legislation policing driving under the influence of alcohol (which has been highly efficacious in reducing some alcohol-related harms), restrictions are often not implemented or enforced for political reasons and have limited feasibility [17]. Education measures such as public information campaigns and warning labels also vary in effectiveness [18]. Persuading behaviour change requires that consuming alcohol is recognised by the individual as being a problem. However, the vast majority (87%) of Australian drinkers consider themselves ‘responsible drinkers’, even though 68% of Australian drinkers consume 11 or more standard drinks on a ‘typical occasion’ [19]. In this way, the majority of people who are drinking over the recommended limits do not regard their consumption as risky or problematic, which leads to alcohol reduction interventions being resisted or even unnoticed [20].

Efforts to reduce risks from alcohol intake often compete with the seemingly valuable aspects of drinking alcohol consumption, a value-base upon which industry capitalise [21]. The sober curious movement and thus, sober curiosity, does not obscure or ignore women’s reasons for consuming alcohol, rather it encourages reflection on those reasons. In our previous work we have explored how alcohol functions as a resource in women’s lives and have identified that these ‘uses’ of alcohol ‘compete with’ public health risk messaging [14]. Research on sober curiosity is relatively new. Promoting the idea of reducing alcohol consumption through more ‘mindful’ drinking is accompanied by an expanding market of alcohol-free beverages, ‘dry’ drinking venues, or licensed bars offering alcohol-free options and increased visibility of these through their endorsement at popular leisure events (i.e., the arts, sport). Sober curiosity not only represents a shift in Australia’s ‘culture of intoxication’ [22], but is also being appropriated by the alcohol industry. In Australia between 2016–2019 the proportion of ex-drinkers increased from 7.6% to 8.9% [23]. Sales of no or ‘zero’ alcohol products (less than 0.5% alcohol by volume) increased by 83% in the 12 months following Australia’s initial COVID-19 lockdown periods in 2020 [24]. Since then, there has been a substantial increase in the supply of alcohol-free wines, beers and spirits (mainly gin), linked to the sober curious movement. Globally, sales of zero alcohol products are surging and are predicted to increase 24% in Australia by 2024 [24]. There is an abundance of research on alcohol reduction from the perspectives of alcoholism and dependency, also legislative or guideline-based approaches, and alcohol refusal, but research on reduction toward moderate consumption as part of a global movement toward wellness, which was prominent during the COVID-19 pandemic, is a rapidly emerging area of interest [25,26]. Research on sober curiosity is relatively new. Ours is the first Australian study to our knowledge that reports empirical data on midlife women’s sober curiosity.

In Australia and other high-income countries, it is young adults who appear to be driving alcohol-free lifestyles and drinking declines [27,28,29]. For example, in Australia research notes a decline in drinking amongst younger people that is attributed, at least in part, to increasing pressure to value and prioritise ‘healthy’ choices and lifestyles and be ‘successful’ and ‘productive’ [30,31]. Similarly, in the UK, research suggests young people feel increasing pressure to ‘hustle’ and achieve success in an increasingly anxious and uncertain social context, leaving little time for the pursuit of pleasure or hedonism drinking alcohol may offer [32]. There is also evidence that moderate drinking or not drinking is becoming increasingly socially acceptable for young people; moving beyond the stigma or judgement that might traditionally be associated with alcohol refusal [33,34].

However, women are over-represented in programs promoting temporary periods of abstinence, such as FebFast (a month of sobriety) or Hello Sunday Morning (an online program where people commit to a period of abstinence and communicate with others about their experiences) [35]. Similarly, platforms and spaces for expressions of sober curiosity—including social media accounts, contemporary ‘quit lit’ and new online communities are mostly run by and used by women [36]. Participation in online sobriety communities fosters an inclusive space for like-minded individuals, offering emotional and social support to others attempting to reduce or cease their drinking [37,38]; this support may be salient for women who may feel under-represented in more ‘traditional’ recovery communities [39]. Studies evaluating participation in these programs have shown that being newly sober provides opportunities for doing identity work [40,41]. Such findings are echoed in other research with those new to sobriety; for example, research with recently sober women living in the UK suggests sobriety is an opportunity to reclaim control and agency over one’s life and present a more ‘authentic’ self [42], particularly for midlife women [43]. Within this research, sober curiosity is framed as a flexible and positive ‘lifestyle choice’; a decision to reduce drinking is promoted as beneficial for everyone and linked to wellness, authenticity, personal growth and improvements of the mind and body [34]. Such conceptualisations move away from medicalised language of addiction, target a specific subset of the population and advocate for complete, lifelong abstinence. This previous research points to the desirable aspects of the expanding sober curious movement for women. However, against a backdrop of ‘healthism’ [44], that is, the increasing moral imperative to take responsibility for one’s own health [30,42] women’s preparedness for sober curiosity—and ability to engage with notions of wellness more widely—continue to be overwhelmingly shaped by social class and this is not always considered. This paper addresses this theoretical gap, contextualising women’s reflections on their drinking practices against the groundswell of a burgeoning wellness industry [45].

## 3. Materials and Methods

Interviews were conducted with 27 Australian midlife women (aged 45–64) and 2 women’s advocacy groups living in Adelaide, Melbourne and Sydney in February and March 2022 by BL. BL was a 39-year-old woman with experience conducting qualitative interviews with midlife women on the topic of alcohol consumption—using techniques of ‘empathic neutrality’ and those akin to life histories which are suggested to increase validity by privileging women’s own subjective meanings [46].

### 3.1. Sampling

Women who consumed alcohol but expressed interest in exploring reducing alcohol (having a ‘sober curiosity’) were recruited through a targeted Facebook advertisement that asked ‘are you sober curious?’, ran for 2 weeks and was released in February 2022. This coincided with ‘FebFast’ and the advertisement was released while FebFast was being advertised online. Initially, 30 women were recruited, but one became unable to participate after contracting SARS-COV-2, another withdrew due to a significant life event occurring, and one was lost to follow-up after completing the social class survey, resulting in 27 participants. In addition to individual interviews, two interviews were conducted with representatives from advocacy groups for women to speak on behalf of women who are single mothers and who live in poverty, this was because of limitations in access to women living with such experiences. To explore the notion of sober curiosity as it relates to class, we sampled for women with access to different levels and compositions of several forms of capital—economic but also social and cultural resources per Bourdieu’s sociological model of class [47]. To measure women’s social class positionings, we operationalised a novel sociological approach recently validated in the UK [48] and Australia [49] and our previous study [13,14]). This approach extends beyond simple economic, employment, and educational markers and has contemporary relevance to the nuances of social class divisions and consumer behaviour that extend to the social and cultural dimensions that shape life chances and alcohol-related outcomes. We have provided detail about the survey tool we adapted from Sheppard and Biddle’s survey of Australian’s social class in 2015 and shown its value for seeing social class in data on women’s alcohol consumption behaviours elsewhere [8]. The survey tool measures social class across three domains: economic capital was measured as income, property and assets, social capital was measured by social contacts and occupational prestige of women’s social networks and cultural capital was measured by the level of women’s participation in various cultural activities. To determine economic capital, assets was measured by combining responses to the questions: what is your annual income before tax or anything else is taken out? (responses were indicated by income brackets provided); what would you say is the approximate value of the property owned or mortgaged by you, and roughly how much do you have in savings? (<$20,000; $20,000 to <40,000, $40,000 to <60,000, $60,000 to <80,000, $80,000 to <100,000, $100,000 to <150,000 and $150,000 or more). Social capital was measured by totaling the number of a range of known occupations within the respondent’s social contacts (i.e., yes = 1) and the average prestige of those occupations. Occupational prestige was assigned using the Australian Socioeconomic Index 2006—a validated index for occupational prestige for the following occupations: secretary, nurse, teacher, cleaner, university lecturer, artist, electrician, office manager, solicitor, farm worker, chief executive, software designer, call centre worker, and postal worker. Cultural capital was measured by a count of “highbrow” and “emerging” cultural activities (where 1 = yes) per Bourdieu’s description of cultural tastes. Respondents selected activities they had engaged in within the 12 months prior to completing the survey from a list of cultural activities including: seen plays or gone to the theatre, watched ballet or dance, gone to the opera, gone to museums or galleries, listened to jazz, listened to classical music (classified as “highbrow”) and listened to rock and/or indie music, attended gigs, played video games, watched sports, exercised or gone to the gym, used Facebook or Twitter, done arts and crafts, socialised at home, listened to rap music (classified as “emerging”).

**Figure 1:**
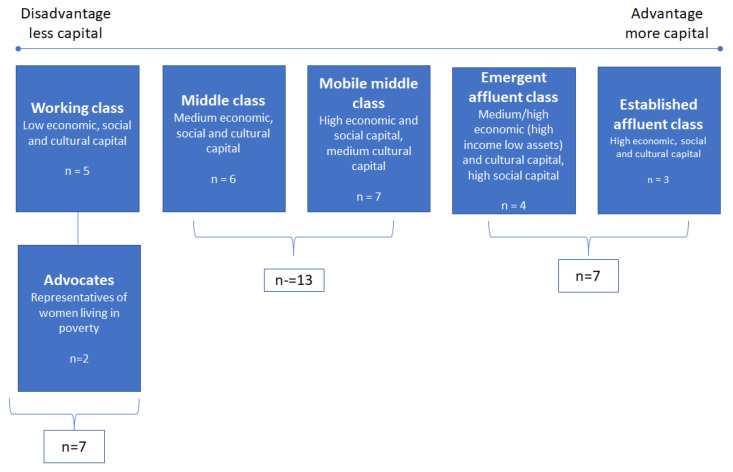
Social class characteristics of sample.

Figure 1 shows the five social classes that resulted and have been collapsed into three classes on the basis of compositions of more or less capital for the purposes of presenting findings: working, middle and affluent:

Women varied in their employment arrangements (full-time, part-time, unemployment, on a pension, retired), their experiences of COVID-19 countermeasures per state-based differences (because women living in Melbourne experienced far longer periods of lockdown in the 12 months preceding the interview than women in other states and one of the world’s longest lockdowns and this may have been relevant to their perceptions of alcohol and health risks [50,51,52]), and their living arrangements (alone, with others including children and/or partner). Some women’s living arrangements changed as a result of COVID-19 lockdowns including relationship breakdown. We recruited women who were alcohol drinkers at the time of the interview and were interested in moderating their consumption or abstaining—i.e., interested in the sober curious movement. Participants self-identified as ‘occasional/light’, ‘moderate’ or ‘heavy’ drinkers—there was some variation here as the main phenomenon of interest was the practices and processes associated with reducing or moderating alcohol consumption (irrespective of the participants’ initial level of consumption or relationship with alcohol). Self-report data was utilised because it captures women’s self-perceived levels of alcohol consumption, which was fit for purpose for our study about women’s perceptions of the possibilities for alcohol reduction that in turn influence their preparedness for sober curiosity. Our sample was mainly Anglo-Saxon although several European migrants participated including one woman from Germany and one from Italy. We did not purposively recruit Aboriginal and Torres Strait Islander women nor women from South Asian or Middle Eastern ethnicities as we acknowledge the specific historical, racial religious and on-going health and social inequities that would require an intersectional study.

### 3.2. Interview Questions and Approach

The interviews were open-ended, lasted on average 60 min and were focused on understanding the contexts that make midlife women interested in sober curiosity or willing to consider reducing alcohol consumption by exploring the factors (practices, intentions, motivations, tools, social networks) that might enable alcohol reductions and allow women to consider reducing consumption. We aimed to explore how and why women’s drinking practices develop, why they persist and how these influences shape continuation or change in consumption patterns. Women’s perceptions about any changes that would enable reductions and what they anticipated gaining or losing from reducing alcohol consumption were explored and also their perceptions of structural enablers and constraints on reduced intake for women ‘like them’. Interviews were conducted and recorded via Zoom, transcribed using Otter Ai and refined by author BL. Only two women did not want to conduct the interview on Zoom (both in working-class positions who expressed discomfort and distrust with online technology) and accordingly, these interviews were conducted over the telephone and recorded via a digital recorder. We did not perceive a difference in rapport or the depth of information collected by using either of the two mediums for data collection because we followed women’s personal preferences. All participants received a $30 shopping voucher to recompense their resources spent on participating.

### 3.3. Ethics Approval

Ethics approval was provided by Flinders University Human Research Ethics Committee. Consent to conduct and record the interview was sought verbally and provided by all women and documented via the interview recording. Pseudonyms are used here to present findings.

### 3.4. Data Analysis

Data were managed using QSR NVivo version 13 data analysis software. Analysis followed a rigorous method of pre-coding, conceptual and thematic categorisation and then theoretical categorisation [53]. Inductive coding was free-hand, paying attention to significant concepts. NVivo 13 was then used for conceptual and thematic categorisation. Using a combination of inductive and deductive logic [54] an initial coding framework comprising open-coding, emerging ideas from literature and media reports on sober curiosity guided coding of all transcripts. Authors BL and PRW discussed themes to check for agreement in coding. Author PRW read data summaries developed by BL and his expertise in social theories of risk and public health was utilised to expand theoretical interpretations and identified areas to improve explanatory rigor—particularly concerning women’s motivations and intentions to reduce consumption, deductively inferring social class differences. Interpretive discussions also took place with author EN who has undertaken research on sober curiosity in the UK. The coding framework was refined accordingly and applied to all transcripts. NVivo 13 was used to generate matrix coding queries across social class attributes and concepts/themes concerning sober curiosity; classed patterning was identified and directed researchers to key excerpts for closer reading. For example, classed patterns were obvious in the coding organised under concepts such as ‘future expectations’, ‘health consciousness’, ‘class identity’, ‘sustainable change’, ‘normalisation of drinking’ and across various positive/negative emotions that were described in the context of not drinking alcohol.

## 4. Findings and Discussion

Two overarching themes relevant to public health understandings of women’s willingness and capability to reduce alcohol consumption emerged through the process of thematic categorisation and the factors that impact women’s sober curiosity including women’s perceptions of the possibilities available to them to not drink alcohol (or drink less) and the circumstances required for them to feel prepared to reduce alcohol consumption. Accordingly, findings are organised by social class advantage and disadvantage. The intent is to distinguish clearly social class-based patterns and differences in women’s sober curiosity. This allows us to readily identify inroads for public health approaches to alcohol reduction messaging segmented by levels of advantage and disadvantage in women’s resources and their life chances.

### 4.1. Affluent Women’s Preparedness to Reduce Alcohol Consumption: Desiring Self-Regulation through Sober Curiosity

Affluent women in our study have given sober curiosity extensive thought, to the extent that several have self-imposed rules governing their consumption levels. For example, Rosie who works in a full-time professional role that she describes as ‘taxing’ and has therefore limited her mid-week drinking says:

*“I had all of these rules about alcohol. And for the most part very successfully, and certainly people’s perception would have been that I was very successful in controlling my alcohol consumption [….] but what I actually felt was that all of these rules meant that alcohol was looming large in my life in a way that just didn’t make any sense to me, like all of the rules actually meant that I was thinking about alcohol all the time.”*

Affluent women’s preparedness to reduce alcohol consumption seems to stem from a desire to self-regulate as part of a perceived need for self-control and health consciousness. Women in our studyrefer to ‘desirable’ levels of consumption, using the drinking occasion or setting as a barometer for gauging what is ‘too much’. For example, Gloria comments on her impetus to reduce consumption as a “*realisation I’m above my weekly target*” and that “*it’s not even ‘special occasion’ [type of] drinking*”. Her sober curiosity is an effort to “*eliminate midweek drinking*”. Sonia expresses dismay at being successful in life but ‘unsuccessful’ at reducing alcohol consumption:

*“I suppose one of the things that I find really interesting about being a person who has an alcohol problem is that I am a kind of a high achiever at a ridiculous rate. So, I find it really hard that this is the one thing I can’t solve” and says: “I suppose it just frustrates me […] I think the best me probably would be better without alcohol or to be able to manage it in a more appropriate manner”.*

Sonia explains she is “*not the only one*”. She elaborates that her girlfriends are “*exactly the same*” as her in terms of their alcohol consumption patterns and perceptions of alcohol-related risks, and remarked upon the “*strong strings the alcohol has over them [all]*”; adding she has “*self-determination in most ways, except that one*”.

Sober curiosity appears to be inhibited by the perceived utility of alcohol consumption. For some it serves as emotional self-management, facilitating “*winding down*” and managing the strains on their time. For example, Penny explains: “*it’s [drinking] just about the fluidity of the moment and (being) able to move with it*”. Affluent women in our study mostly occupy full-time paid work roles in professional careers and some describe being at the “*peak of my career*”; they said they feel their work is demanding and rewarding and it is integral in their personal and social class identity. Affluent women use words like “*hectic*”, “*exhausted*” and “*achievement*” frequently and often in unison; and as they rationalize alcohol consumption or as Penny says explain how it “*takes the edge off*” when you’ve been “*on the rails all week*”.

For Gloria, sober curiosity competes with the value of alcohol and the ritualistic aspects of a drink when “*switching off between work and home*” and creating “*headspace from work or demarcating relaxation time*”. She remarks that sober curiosity would be possible if she “*switched the narrative slightly to be away from the default position of the glass of wine to an alternative*”.

Beneath the seemingly simple desire for self-regulation, it seems Gloria, and affluent women like her, are cognisant of more complex social expectations regarding respectable alcohol-related behaviours; she describes feeling negative emotions when expectations are unfulfilled:

*“myself and probably quite a lot of my friends who are very competent, educated, high functioning people, that kind of shame does [factor in], I feel really ashamed that I can’t get more on top of my drinking, because I feel like I’m pretty on top of most things in my life. But I do have … this sense of shame and that’s the one thing I just can’t seem to really get on top of. I feel a lot of shame about it, which is interesting …it gives me far more than it takes so it’s really hard to…and it’s all I’ve thought about doing [drinking].”*

Part of this shame would be relieved through alcohol reduction, which most of the affluent women explain feeling it would allow them better interactions and relationships with other people. Several of the affluent women who are mothers, such as Bronwyn, describe complexities with parenting teenagers or young adult children and concerns about “*increasingly anxious young people” and* that reducing alcohol would allow for good role modeling to their older children and relief from the burden of worry about their older children. However, they also describe feeling “*peer pressure*” to drink when away on holiday with girlfriends which as Bronwyn explains “*leads to excessive consumption*” and as Gloria comments, leads to moments of feeling “*regretful I crossed the line*”. Our analysis suggests reducing alcohol offered women a chance to cope with shame that they had failed in proper self-management at work or in their role as a mother. In some instances, the shame seemed to manifest in women trying to manage the contradictions and tensions that ‘too much’ alcohol consumption has with their identity as a ‘good mother’ [55,56]. For example, Bronwyn comments: “*I wouldn’t tell most of my friends how much I actually drink*”; whereas Elizabeth describes a tolerance not necessarily encouragement to drink excessively amongst her friends: “*we drink a lot and it’s very accepted*”. Both instances point to the possibility for alcohol reduction.

For affluent women, the general need for self-preservation was part of the value of drinking alcohol in the first place; as Ellen explains when she considers what she would lose if she reduced alcohol: “*you [would] lose the ability to hide and push things back*” the “*ability to be able to shut myself down and relax*”. Affluent women also spoke of feeling that their struggles were invisible: Elizabeth explains “*we need recognition and to be recognised*” referring to a lack of recognition for her achievement of juggling multiple competing family responsibilities and career success in a role where gender bias exists. The role of alcohol as a stand-in acknowledgment did not give women visibility but fulfilled their need to cope and this is evident in Sonia’s comment:

*“it’s also because they’re insanely busy. But then there is the sort of high functioning anxiety that comes with that busyness and that giving to everyone…” and “I have a lot of girlfriends who are about my age. So, in their 60s […] we’re not really terribly alone in the sense that a lot of people who are drinking about a bottle of wine a day who really shouldn’t be they’re intelligent, capable and wonderful women who, for whatever reason, have been self-medicating [by drinking alcohol]”.*

In such instances, affluent women like Ellen describe a flipside of reducing alcohol would be adding to her existing mental load: “*it would cause unnecessary strain to completely abstain which requires management and would induce more stress.*” Contemplating reducing alcohol clearly can feel like a lot of ‘work’ for affluent women when managing the ‘mental load’ in their lives, which is one of the reasons they gave for consuming alcohol in the first instance.

For some affluent women the benefit of alcohol is felt so acutely it is perhaps impossible to see drinking as a problem; such as Penny who says “*the motivation hasn’t been strong enough to lean that way*” and “*I don’t see alcohol as a problem*”; the general sense was that giving up alcohol would need to result in a tangible, noticeable and worthwhile outcome for women. Bronwyn remarks that the benefits of alcohol reduction would have to be “*really dramatic*” in order to make the strain required to abstain “*worth it*”. Affluent women perceive the idea of going without alcohol and completely abstaining “*confronting*” (Ellen) and sober curiosity appeals because they could retain a sense of agency and control and this seems to feel gentler; “*you don’t have to give up all together, you can try and cut back*” (Ellen). Affluent women want assistance to achieve “*sustainable long-term change*” (Rosie) and “*a good quality of life as long as possible*” (Gloria). Others feel dissonance with the religious and philosophical stance of programs like AA or simply they cannot ‘see themselves’ as represented within or suited to the program (an online app or social media account). A peer-support type network emulating current programs that have been successful for other population groups but purposed for midlife women is possibly suitable. Sonia suggests:

*“I wonder whether something like the [name of alcohol sobriety program], but geared toward women in midlife, and their reasons for consumption, the supportive platform like that might be a way forward because from the women I speak to, they are looking on social media. And I know I wasn’t sure I do. Are you on Instagram? Everything almost everything they’ve got is on social media …. [it could be a] very effective platform for us.”*

Bronwyn’s narrative shows agreement with Sonia, and she recommends adaptations to the goals of the program to suit affluent midlife women’s lived experiences:

*“I talk about this a lot with my friends, a bunch of extremely busy educated women with sort of still crazy lives…we talk about it quite a lot…. quite a lot of my friends are in the same situation they realise they’re probably drinking too much…dependent on alcohol. Use it as a bit of a coping mechanism in life. I think sometimes then when you go down the [mentions alcohol sobriety program]… I just actually got so sick of reading these stories of women who say how their whole lives are transformed when they stopped drinking. I just stopped reading the articles halfway through, because I’m like ‘no, that’s not me’, I don’t want to totally stop drinking and alcohol isn’t destroying my life, but I am probably drinking too much alcohol. I like this idea [sober curiosity] more around actually being curious, and thinking about maybe reducing, moderating, being very mindful that you’re drinking, but not saying, my goal is to never drink again”*

(Bronwyn).

Most affluent women in our sample felt it would be “*appealing and desirable to go somewhere you can be a part of the normal*” (Gloria) warranting the assimilation and normalisation of reduced consumption within women’s social and leisure spaces.

### 4.2. Middle-Class Women’s Preparedness to Reduce Alcohol: Sober Curiosity as Civility and Respectability

Within our data, sober curiosity seems the most possible for middle-class women; for a multitude of reasons. Reasons include women’s concerns with the sustainability of drinking patterns (specifically an increase in the frequency of consumption) they had established during COVID-19 lockdowns and a realisation that the stresses that lead to drinking alcohol in order to cope have not gone away because COVID-19 continues to impact their lives: “*COVID is going on for so long that it’s [drinking a lot] is not sustainable*” (Kelly). Sober curiosity arose through experiences that resulted in feeling like alcohol takes more than it gives “*alcohol as ‘giving’ is fiction*” (Angie) and feeling boredom with daily living and looking for a personal challenge: “*the drinking has become mundane, not drinking is a new challenge*” (Mandy). Some women link sober curiosity to the lifecourse and feeling “*ready for a new phase of life*” (Kelly) evident in statements such as “*I am developing an awareness of my own mortality*” (Sonia) and descriptions of non-drinking as “*identity work that people are experimenting with*” (Heather). The middle-class was the only social class group where women mention the lifecourse from a physiological perspective and women’s sober curiosity is encouraged when they felt reduced inflammation or hormonal disregulation by not drinking alcohol: “*the menopausal symptoms are better without alcohol*” (Pamela) and “*menopause and alcohol is a really bloody hard combination*” (Raven).

Civility and notions of respectability are key themes that emerged through our analysis of middle-class women’s preparedness for alcohol reduction. Compared to affluent and working-class women, middle-class women speak about sober curiosity with undertones reminiscent of neoliberalism, particularly prominent in their individual responsibilisation for drinking to ‘excess’ and therefore, for making reductions. Several middle-class women’s narratives suggested reducing alcohol is a sign of personal strength and resilience for example, Alison says: “*having a drink is nice to do when you’re pretty wound up and weak*” and Heather expresses feeling “*more disciplined and structured*” and that moderate drinking is “*a standard [I] want to set*”.

Even where social influences and cultural acceptability of alcohol consumption are acknowledged, middle-class women link limitations in the possibility of sober curiosity to personal motivation. For example, when asked about the factors that make reducing alcohol (im)possible Kelly responds:

*“Tough to know. I mean, some of it is socialising. I know that, because I’ve got friends who drink, family…they’ll drink. So some of that will be that. So, who you are socialising with what you’re doing. So that’ll be fun, you know, some friends particularly probably. Family somewhat… Cause I’ll just be drinking. I think ‘I’ll just have one more’ and I’m enjoying that taste and then you don’t stop. So they’re probably the main things… almost feels like willpower, really”.*

Some middle-class women’s logic for sober curiosity represents dutiful ideals and, in several instances, censure of ‘irresponsible’ behaviour. For example, Alison feels herself personally responsible for “*breaking the chink in the chain of alcohol*” and to “*establish new patterns for ourselves*” when she comments on Australia’s heavy drinking culture. Alison says she feels “*affronted by the (drinking) culture and participating in that*”; she explains that this influences her preparedness to reduce alcohol consumption. Concerningly, Angie describes this personal responsibilisation for reductions as “*laborious*” and she feels it results in women ‘sneaking’ alcohol and then justifying it as a valid reward—she describes feelings of guilt and sadness surrounding her ‘failure’ to moderate alcohol. Ruth explains her sober curiosity in the context of explaining the social popularity of heavy drinking amongst her peer network (both close friends and more distanced peers such as colleagues and school or sport parents) she is willing to be “*going against the grain*”. She is familiar with the alcohol guidelines for ‘moderate’ consumption in order to reduce health risk and remarks “*that’s what I will stick to*” adding that “*I’m not afraid not to be cool*”; she connects this to personal strength and remarks “*I’m strong enough [to reduce alcohol]*”. Her preparedness for sober curiosity seems motivated by personal responsibility to reduce drinking and she uses words such as “*destroy*” and “*sabotage*” to describe drinking to excess and wants to avoid those negative experiences ‘for and by herself’. Ruth does acknowledge that mental health options are unavailable or under-utilised by women she knows and that alcohol’s role as ‘self-care or self-medication’ makes sober curiosity less possible for them:

*“The women that are kind of using it as their medicine of choice, rather than getting mental health plans or whatever…they will fight you tooth and nail: ‘No, no, nothing wrong with me ‘you know, like, ‘No, no’, they will not take mental health medication or seek the help or maybe it’s too hard to get the help. So, they get out now just go get a drink. Alcohol works… that’s like a chemist for them”.*

Alison explains that for her, sober curiosity is possible because of “*a social network that allows it*”. For other middle-class women such as Pamela, reductions are only possible by “*flying under the radar and not making a scene of it*”; among her social network she feels people hassle non-drinkers because they want their own drinking normalised. Certainly, middle-class women observe heavy-drinking norms among women like them, for example Mandy comments on the culture of drinking among mums that she feel ‘glamourise alcohol’. Joanne comments that it was difficult for her to feel part of her social network without drinking:

*“I often think afterwards, I didn’t have the same feeling of having been fulsomely in the social situation when I’ve been not drinking… I have found that it hasn’t felt the same kind of authentic socialising.”*

Alison feels that “*focusing on alcohol would expose a personal failing of not coping*” potentially conveying to others that you are not an ‘authentic’ midlife woman and mother who fits the ‘right’ stereotype. While on a similar theme of acceptability and expectations, Nancy remarks “*my partner is resentful if I don’t drink*” because drinking is something she feels they do together that he thought symbolises to her partner that she is relaxed. It seems alcohol reduction and interest in sober curiosity would each be more possible for middle-class women if their use of alcohol as a stand-in for absent support was taken seriously rather than joked about within a socially accepted culture of drinking; which women express ‘plays down’ the seriousness of their emotions and their emotional needs in relation to alcohol.

Nancy says she is seeking options that are “*wholly relaxing*” not just “*momentary*”—“*alcohol is very momentary*” and there is an absence of alternatives. Raven describes feeling she is living in an “*invisible age*” in terms of appropriate support, she feels there is suitable and purposeful mental health services for young people and there is a group chat in mental health prevention forums that would be good for midlife women so she “*wouldn’t feel so alone*”. Alongside this, alcohol is so readily available, she says: “*it can easily get into the house*” and she spoke about having used alcohol home delivery services. Raven feels it relieves some of the burden of her caring role when she “*couldn’t leave [her] parents*” and describes feeling “*hypervigilant*” and says she was drinking in order to cope with the feelings of constant burden from caring. Our findings also suggest middle-class women have the affordances of resources to participate in periods of intermittent sobriety or ‘fasts’ (e.g., FebFast, Dry July and Sober October). Middle-class woman Nancy feels this offers a defined period of time (and thereby a sense of control and possibility), where no one questions alcohol abstinence and the philanthropic pursuit attached to fasts is considered a noble thing.

### 4.3. Working-Class Women’s Preparedness to Reduce Alcohol Consumption: Complexities and Impossibilities for Sober Curiosity

Many working-class women in our sample describe feeling ‘scared’ of what life without alcohol would be like; quite a distinct difference from the narratives we heard from more affluent women who describe reduction as difficult to achieve but not impossible. For working-class women, reducing alcohol seems particularly complicated, and our analysis reveals the breadth of the value of drinking for such women that extends from and also beyond the intoxication of the drinking occasion into recovering the day after. When we consider working class women’s preparedness for sober curiosity, we realise the deep layers of oppression that need to be peeled away before alcohol reduction can become a possibility, particularly for women living with considerable disadvantage let alone something they feel prepared to do. For example, Barbara describes feeling a “*deep seated loneliness*” and explains she lives alone in a caravan park where she is unable to house a pet for company. The possibility for reducing consumption hinges on her finding happiness and confidence outside of alcohol: “*I just want happiness and to be able to just go and do things and not need a drink to make me happy and outgoing*”. Alcohol consumption for each of the working-class women seems a key form of enjoyment (sometimes the main or only form), as Celeste remarks: “*alcohol is an only form of fun or interest…something to look forward to*” and adds “*we don’t get many other opportunities to feel carefree*”. Sober curiosity would reduce working-class women’s chances for reprieve from their hard lives, and would require a presence when the desire and need is absence or distance from the difficulties of life, such as Barbara: “*I’m drinking just to take me away from everything*” and “*I’m drinking to numb negative thinking and horrible stuff*”. This poses a crucial barrier to preparedness and stifles working-class women’s possibilities for sober curiosity. The working-class women in our sampledescribe worrying about feeling exposed by having nothing to do and this worry manifests in alcohol consumption, as Barbara explains:

*“I get lonely and I get bored. I lost a job back a year and a bit ago because I turned up at work smelling of alcohol and I have had more jobs since…. I drink alcohol like water and I was getting to the stage where I was drinking to combat the after effects of drinking that has been hair of the dog for [my] hangover”.*

The possibility for sober curiosity seems non-existent in this continual and ‘necessary’ engagement with alcohol. Recovering from the night of drinking and ‘nursing a hangover’ seems to give some working-class women (without paid employment) something to do; something to manage in the absence of another plausible way to occupy time—certainly, the hangover is a valid if not sometimes revered experience in Australia’s alcohol saturated society (its telling of a ‘big night’ spent drinking), perhaps more valid than having nothing to do, as Mary explains “*at least you have a hangover*”. Perhaps a hangover offers working-class women a distraction from the bleakness of life and its ‘daily grinds’; certainly, Helen expresses concern about her own self and women like her: “*what would life look like if we weren’t hungover*”. Mary explains: “*I worry about what I would do with my time if I didn’t have a hangover as an excuse*” and “*the alcohol numbs and the hangover provides a distraction*”. Having a hangover is spoken about in ways we interpreted as a productive means of demonstrating agency and regaining control: “*actively engaging in disentangling yourself from real life*”. It seems that an extension of the numbing effect of inebriation is the dull headedness of the hangover, and both are coping strategies. It follows that limits in possibility for sober curiosity among working-class women, were limits in their preparedness for alcohol reductions.

Unlike affluent women who could identify avenues for support for more moderate drinking, albeit not directed at their age-group, working-class women cannot conjure up such support; as Celeste explains:

*“I think that’s one of the things is you get scared about doing this, because you think I don’t want to give up alcohol for the rest of my life. And I think it’s […] an either/or—either you drink or you give up completely. That’s where I want to find that medium. Where do you talk to likeminded people who actually want to achieve that? Is there a group that you can actually say, ‘This is what I want to achieve?’”.*

The alcohol reduction programs that affluent women feel could be tailored to them cost money and this precluded participation among less well-resourced women: “*we need to make free the ones that sell you stuff and say the perk is reducing your money [spent on alcohol]*” (Barbara). Some of the working-class women we interview describe feeling surveilled once they had searched for internet sources of alcohol reduction support, and prefer the idea of phone consultations, Celeste says:

*“I don’t think I’ve necessarily found something that is just about trying to change my drinking a little bit […] what I find interesting is obviously, everything’s monitored, so you might search for that [reducing alcohol] and then all of a sudden, I’m getting all these adverts for basically how to deal with being an alcoholic and it’s like, no, no, I’m not saying I’m an alcoholic. I certainly have a relationship with alcohol I don’t need all that self-help stuff. So, I’ve not quite found anything yet that’s supportive of women without it being we’ve all got a problem here sort of thing”.*

An advocate speaking on behalf of women in poverty delineated the layers of complexity experienced by working-class women living on very low incomes (if not in poverty) for accessing suitable support for mental wellness in order to feel prepared to reduce alcohol consumption:

*“to get counselling, depending upon your age, you might need to go to a doctor and have a mental health plan and then you have X amount of sessions, and it’s never free. It’s just at a reduced cost. Then if you know of some community services, you need to actually do a whole lot of introductory work before you can even access that service. So it’s a lot of cost and emotional work, and a willingness to put yourself out there before you can find any sort of level of support.”*

She also sheds light on the limitations for social participation and the need for peer support opportunities that acknowledge the limitations working-class women face:

*“the ability to share with your peers… when you’re engaged in a professional network, or you have a nice social network, you can bounce around what’s happening to your life in a trusted supportive space with like others. But if you’re isolated, or you’re trying to pretend in a group…you start to isolate yourself away”;* she added *“there’s a whole lot of reasons why isolation, based on money is a real factor”* and that we need to *“create a way for people [women] to speak and make sure that there are different sorts of support networks for them”.*

Another advocate reminds us that working-class women are also often single parents and this means if they spend time engaged in self-care, their full time ‘position’ as domestic labourer and carer needs to be ‘backfilled’. Having a drink of alcohol as a stand-in support requires no engagement in any of these resource intensive and emotionally intensive processes and so this needs to be factored into reduction possibilities for working-class women. The timing of delivery of risk reduction messaging is also crucial, in the current economic climate: Australian women on low incomes are dealing with inflation in the costs of living and interest rates increasing if they have borrowed money. As one of the women’s advocates remarks: “*why the hell would you give up [drinking] now?*”

### 4.4. Class-Segmented Approaches to Alcohol Reduction for Women

Social class differentiated the factors that influenced women’s preparedness for sober curiosity and, most critically, were the contextual and social class frames for their drinking. More affluent and middle-class women discussed a desire for self-regulation and ‘proof of willpower’ as motivations for sober curiosity; most often this individual goal was nestled within a deeper social context comprising gendered social norms, where they felt personal responsibility to control the direction of their lives even when circumstances were outside of their control. Our analysis suggests drinking alcohol is a means of managing social expectations, or for coping, for which women internalised responsibility and therefore felt drinking was something they felt they ‘should’ personally manage. For example, women with more advantage felt that alcohol consumption rather than moderate or non-drinking options is normalised in their social contexts (both face-to-face and online Zoom drinking sessions) and are practices from which they had positive experiences and gained social and cultural (social class position affirming) capital. For women with less advantage, who consumed alcohol to manage difficult lives and negative emotions, where alcohol consumption was less embroiled in socialisation and more so part of daily coping, preparedness for sober curiosity was particularly limited. Extrapolating from our findings, below are options for alcohol reduction targeting the social and cultural contexts of consumption that is – the things that lead midlife women to drink or feel prepared to reduce drinking rather than women’s individual drinking routines or habits – segmented by levels of disadvantage. Options draw attention to and extend from the social factors and contexts that shape women’s possibilities for sober curiosity, and most arise from midlife women’s own ideas put forward during the interviews.

### 4.5. Drinking Culture and Social Expectations

The social settings women occupy that typically feature alcohol contain various avenues for supporting sober curiosity. Affluent and middle-class women who recognised aspects of social acceptability and socialisation in their social class responses to alcohol reduction possibilities noticed a general absence of non-alcohol options in settings where consuming alcohol is normalised. An obvious avenue for change is creating women’s social events or themed activities that do not involve alcohol. Current available options are often alcohol themed or sponsored by the alcohol industry or partnered with alcohol products. Increasing the visibility of these events and alternatives to alcohol in media marketing to women would support preparedness through demonstrating the social acceptability of sober curiosity for midlife women. This would directly respond to women who noticed a general absence of modelling of midlife women non-drinkers, and commented that sober curious social media influencers who are in the same phase of life would be helpful to normalise non-drinking among midlife women.

### 4.6. Increasing Support

Common to all the women in our studywas an absence of support for mental wellbeing but for different reasons on the basis of social class. For affluent and middle-class women, feelings of stigma and shame exacerbated mental instability and conflicted their feelings around admitting they were not coping with the struggles of multiple demands and this reduced preparedness for alcohol reduction. Our findings suggest that for affluent women, improving the availability of accessible support services such as an online forum (women commented on feeling time poor) and tailoring them to be sites where women can debrief with other women and seek solace. Our findings differ from previous research [20] in revealing that some affluent women do see their alcohol consumption as a ‘problem’ and this allows an openness to sober curiosity. According to the affluent women in our study, traditional sobriety programs designed to assist ‘problem’ drinking require a complete overhaul and felt that the sober curious movement is a ‘softer’ and more appropriate option. It would increase their feelings of social engagement and connectedness, allowing them to have open dialogue with other women about struggles that result in alcohol consumption in turn increase their preparedness for alcohol reduction. For middle-class women, making mental health care more available and accessible and particularly, reducing the ‘mental load’ caused by encouraging women to ‘consider their drinking’ through campaigns that ‘responsibilise’ women is critical, because we know that while the normalisation of heavy consumption and women hiding consumption levels beneath dark comedy like termagants continues to occur, and perhaps this is why women talk about the tensions they feel between drinking in order to be social and reducing alcohol consumption in order to manage health risks. For middle-class women, social media has a use-value for sober curiosity and could feature influencers as ‘peers’ who are reducing alcohol and are in the middle phase of life (not young women). For less privileged women, suitable support groups where women are not tasked with quitting in the absence of other structural or emotional supports might be helpful. Interventions should be designed to reduce feelings of surveillance and be mindful not to exclude women because they are ‘unable to invest in themselves’. Women on low incomes may be resource-poor in particular ways that make stopping or even reducing drinking feel difficult to imagine or contemplate. In formulating reduction options, consideration of the resources required is warranted, they need participation which requires trust, safety, literacy and confidence to explain what they are feeling and needing and to feel heard and supported. Women feeling shame about failing to embody the ‘proper’ level of constraint and self-control over their alcohol consumption (gendered shame) is exacerbated for working class women by the shame that seemed to result from their stigmatised social class position.

### 4.7. Limitations and Areas for Research Extension

We cannot be sure about how differences in women’s life circumstances alongside their social class might increase their preparedness for sober curiosity; several women spoke about distancing themselves from parental heavy alcohol intake and one woman mentioned smoking cannabis as her substitute for alcohol. Further exploration of how women’s sober curiosity is shaped by differences in factors in addition to social class is a limitation of this study but seems like a relevant point of inquiry for future studies.

## 5. Conclusions

This study represents innovation in public health research to explore how current trends in popular wellness culture toward ‘sober curiosity’ and normalising non-drinking or lighter drinking and increased health consciousness could increase women’s preparedness to reduce alcohol consumption. It offers insight into how we can drive public health change effectively to reduce population level alcohol harms via reducing alcohol consumption among midlife women, and with sustained impact. Our findings reinstate the importance of recognising social class in public health disease prevention; validating that socially determined factors which shape daily living also shape health outcomes and this results in inequities for women in the lowest class positions. In this case, the inequities are unequal opportunities to reduce alcohol and reduce alcohol-related risks. By exploring modifiable alcohol consumption practices through women’s social class rather than focusing on individual consumption patterns (which is proving ineffectual), we have provided ideas for structurally improving the conditions that would allow all women to feel prepared for sober curiosity. If translated into tailored social class-based options to support midlife women’s sober curiosity, we can plan health interventions that are realistic within women’s life contexts and therefore more likely to have a meaningful impact—and progress toward achieving equity in the reduction of population-level alcohol harms.

## Data Availability

Data summaries can be provided upon reasonable request from the authors.
